# Increasing river floods: fiction or reality?

**DOI:** 10.1002/wat2.1079

**Published:** 2015-03-11

**Authors:** Günter Blöschl, Ladislav Gaál, Julia Hall, Andrea Kiss, Jürgen Komma, Thomas Nester, Juraj Parajka, Rui A. P. Perdigão, Lenka Plavcová, Magdalena Rogger, José Luis Salinas, Alberto Viglione

**Affiliations:** ^1^ Institute of Hydraulic Engineering and Water Resources ManagementVienna University of TechnologyViennaAustria; ^2^ Institute for Systematic Botany and EcologyUlm UniversityUlmGermany

## Abstract

There has been a surprisingly large number of major floods in the last years around the world, which suggests that floods may have increased and will continue to increase in the next decades. However, the realism of such changes is still hotly discussed in the literature. This overview article examines whether floods have changed in the past and explores the driving processes of such changes in the atmosphere, the catchments and the river system based on examples from Europe. Methods are reviewed for assessing whether floods may increase in the future. Accounting for feedbacks within the human‐water system is important when assessing flood changes over lead times of decades or centuries. It is argued that an integrated flood risk management approach is needed for dealing with future flood risk with a focus on reducing the vulnerability of the societal system. *WIREs Water* 2015, 2:329–344. doi: 10.1002/wat2.1079

For further resources related to this article, please visit the WIREs website.

## INTRODUCTION

**Figure 1:**
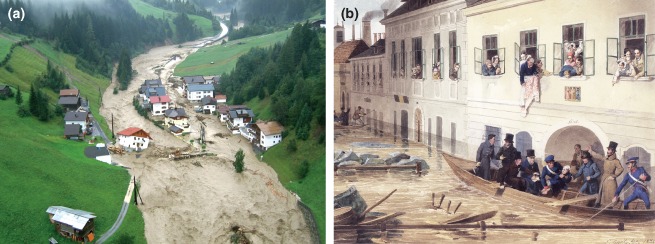
Two examples of river floods: (a) rain flood in Paznaun, Tyrol, August, 2005 and (b) ice jam flood in Vienna, Austria, March, 1830.

Floods occur when a piece of land that is usually dry is submerged under water. There are several types of floods that differ by the processes that produce them.^1^ Coastal floods may be triggered by earthquakes in the ocean. They are called tsunamis such as those that occurred in Indonesia in 2004 and in Japan in 2011. Coastal floods can also be triggered by strong winds in association with particularly high tides. River floods, however, occur along small or big rivers and are usually triggered by rainfall, sometimes in association with snowmelt. An example of such a flood caused by rainfall is shown in Figure 1(a) that occurred in Tyrol, Austria, in August 2005. A particular type of river flood are ice jam floods when, after a cold spell, the river ice breaks up and the ice floes get congested to form a dam that blocks the flow of water thus causing inundations (Figure 1(b)). Another type of floods is dam break floods. A famous example is the Johnstown Flood that occurred in May 1889 and was the result of the failure of the South Fork Dam in Pennsylvania, USA. Such floods may also occur when the dam containing a glacial lake fails. Finally, flash floods are caused by short, small‐scale, but very intense rainfall and may occur in any part of the landscape. If they occur in mountain areas, they are often associated with landslides and debris flows. A recent example is the Cinque Terre floods in October 2011 in Northern Italy.

There has been a surprisingly large number of major floods in the last years around the world,^2^ which suggests that floods may have increased and will continue to increase in the next decades. Recent major river floods in Europe include the May/June 2013 flood at the Elbe and Danube, the May 2014 flood on the Balkan, and the winter 2013/2014 floods affecting much of the UK. However, it is not fully clear whether this perception of universally increasing floods is borne out by observational data. This study aims at shedding light on this issue. It is specifically concerned with river floods that are the result of rainfall and snowmelt. The article will first briefly explain how floods can be measured (Chapter 2) and then examine whether floods have increased in the past in Europe (Chapter 3), review the processes that cause changes in river floods at present (Chapter 4) and finally discuss whether floods are likely to increase in the future (Chapter 5).

There are a number of quantities that are of interest when trying to understand how big a flood is and why it has occurred. The most obvious is the flood water level. Often, one is not only interested in the water level but also in how much water passes through a cross section of the river. This quantity is termed runoff or discharge and has units of volume per time. Rain and snowmelt induced river floods occur when the runoff is much higher than usual. In the remainder of this study, runoff will be used as the main quantity to characterise floods. This is a particularly useful measure as it makes the magnitude of floods more comparable irrespective of the cross‐sectional area of a particular river reach. Specifically, the highest runoff of a flood (termed peak runoff) is an important quantity representing the magnitude of a flood.

## HAVE FLOODS CHANGED IN THE PAST?

**Figure 2:**
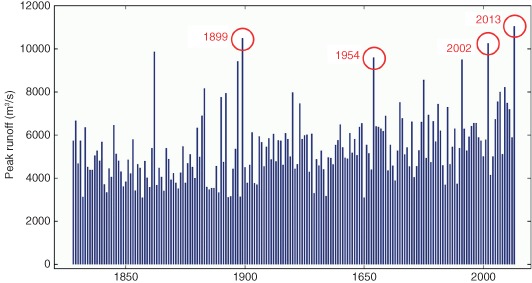
Maximum annual floods (i.e., the largest runoff in every year) for the Danube at Vienna between 1828 and 2013. (Reprinted with permission from Ref 3. Copyright 2013). Publisher is Österreichischer Ingenieur‐ und Architekten‐Verein.

The most objective way of exploring whether floods have become bigger over the past decades for a particular river is to analyze long data records of flood runoff. An example of such a record is presented in Figure 2, which shows the maximum runoff in every year between 1828 and 2013 for the Danube at Vienna, Austria. The graph indicates that there are years with relatively small peak runoff (which one would not actually call a flood) and there are years with very large runoff which certainly qualify as floods. Four of the biggest floods have been marked by red circles in the figure. These are the floods that occurred in June 2013, August 2002, July 1954, and September 1899. All of these floods were produced by heavy rainfall with maximum rainfall rates of over 300 mm (equivalent to L/m^2^) in a couple of days. The exception was the flood in September 1899 where rainfall was much higher (more than 500 mm), yet the peak runoff was not very different from that of the other events. This was because the landscape was rather dry in September due to low summer rainfall. Much of the event rainfall therefore infiltrated into the soil, so the actual runoff from the land surface was somewhat reduced.

**Figure 3:**
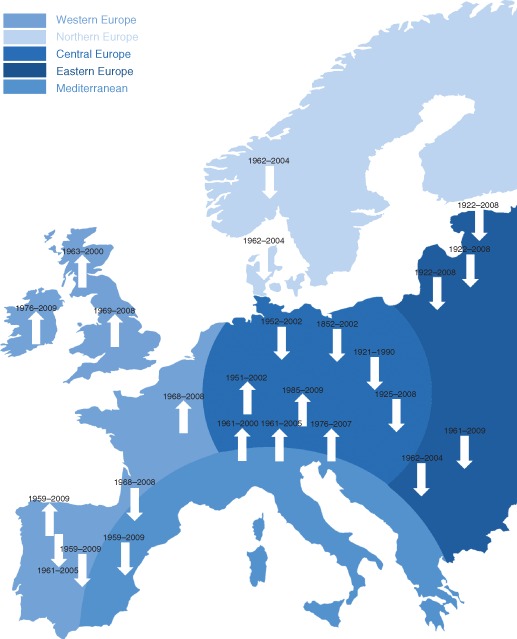
Summary of trends in flood peak runoff in the last decades in Europe obtained from different studies and study periods. Upward pointing arrows indicate increasing trends and downward pointing arrows represent decreasing trends. Both are related to the majority of trends in a region. No arrows are shown in areas with inconclusive data and/or results (Reprinted with permission from Ref 2. Copyright 2014). Published by Copernicus Publications on behalf of the European Geosciences Union.

Plots such as that shown in Figure 2 have been analyzed by numerous researchers around the globe to understand any changes in the flood magnitudes and frequency that may have occurred in the past decades.^2^, ^4^ Often, the interest resides in whether a trend has occurred. To test the existence of a trend a regression line is fitted to the data and it is also evaluated whether that trend is statistically significant. Overall, these studies have shed a lot of light on river flood trends around the world in the past decades but the data show an immense amount of spatial heterogeneity. The heterogeneity is mainly due to local processes that affect the magnitude of floods (as discussed in the next chapter). There are also interesting large‐scale patterns brought out by the analyses. The literature review of Hall et al.^2^ suggests that there was a tendency of floods in the Iberian peninsula and in North‐Eastern Europe to decrease (the latter due to the influence of earlier snowmelt) and a tendency of floods in Western Europe to increase (due to changes in precipitation) (Figure 3). It should be noted that the results of the trend analysis invariably depend on the observational window of the flood series. Figure 2, e.g., would give a decreasing trend if only the period 1890–1950 were considered, but an increasing trend for the period 1950–2013. Differences in the observational window will therefore introduce uncertainty in the analysis.

**Figure 4:**
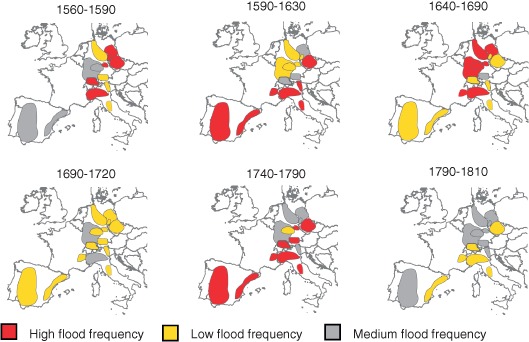
Periods with frequent and less frequent flooding in Europe for selected periods between 1560 and 1810 (Reprinted with permission from Ref 10. Copyright 2010). Published by Copernicus Publications on behalf of the European Geosciences Union.

Runoff observations on most rivers have only started in the late 19th century or even later. It is also of interest to go back further in time to understand flood changes on a long term basis. Such changes can be explored by historical hydrology based on a variety of source material, mainly from the past 500 years, and sometimes even longer. Documentary sources include individual records (e.g., narratives such as chronicles, newspapers, private and official correspondence, and pictures) and legal‐administrative materials (e.g., account books and taxation records). 5, 6 The information provided may include the time and date of the floods, their meteorological and hydrological causes, human losses and material damage as well as the societal responses. Flood marks along the river banks (usually on buildings) provide information about the highest water level of a flood. Both documentary sources and flood marks can be used to infer the flood peak runoff by mathematical modeling.^7^ If one intends to go further back in history, paleohydrology may provide useful information on the magnitude of floods through their environmental effects such as slackwater sediment deposits or scour lines.^8^, ^9^ An example of a European overview derived from historical documentary‐based long‐term reconstructions is shown in Figure 4. The red colors indicate regions and periods where the flood magnitudes were particularly high and/or the floods occurred particularly frequently. An interesting finding of these long term analyses is that floods tended not to occur evenly distributed throughout history but in clusters or periods that were particularly prone to flooding resulting in flood rich and flood poor periods. For example, a flood‐rich period is found in the second half of the 18th century where a great number of large/destructive floods occurred in many parts of Europe (Figure 4, centre bottom). There have been various interpretations of the occurrence of flood rich periods that are usually related to the recurrence of similar combinations of flood generating processes in the Earth System.^11^, ^12^, ^13^, ^14^, ^15^

**Figure 5:**
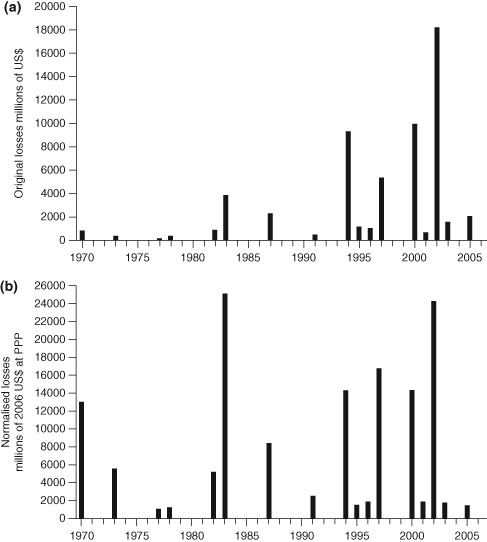
Annual flood losses in Europe from major flood disasters: (a) raw data and (b) data corrected for inflation as well as for changes in population and wealth (Reprinted with permission from Ref 17. Copyright 2009). Published by Copernicus Publications on behalf of the European Geosciences Union.

From a societal perspective one is not only interested in changes in the flood magnitudes (e.g., measured by changes in the peak runoff) but also in their effect on the economy, in particular the economic losses incurred by floods.^16^ One such analysis of the changes in flood losses in Europe is shown in Figure 5, which compares two cases for illustration. Figure 5(a) shows the raw losses in Million US$, i.e., without accounting for inflation or any other changes in the socioeconomic system. Figure 5(b) shows the losses normalised not only by inflation but also for changes in population and wealth. The comparison illustrates that care needs to be taken in interpreting time graphs of losses. Both floods and socioeconomic parameters undergo very significant decadal dynamics. In many countries around the world, the value of assets in flood prone areas has increased enormously in recent years and migration into flood prone areas has also been observed.^18^ While floods have changed in a complex manner, depending on their influencing factors, in many regions the socioeconomic conditions have changed even more.^19^

## WHAT ARE THE DRIVING PROCESSES OF CHANGE?

The processes controlling river floods triggered by rainfall and snowmelt can be grouped into three compartments. The first is the atmosphere where rainfall is produced and the energy conditions for the land surface associated with snowmelt and evaporation are defined. The second are the catchments (i.e., the land surface, the soils, and the groundwater aquifers) where the rain water (and any snowmelt) flows off on the surface or infiltrates into the subsurface. The third are the river systems where the runoff generated locally is transported downstream collecting inflows from an ever increasing number of catchments. The processes in all three compartments affect the characteristics of river floods. Therefore, any changes in these processes will also lead to changes in the floods themselves.

Table 1 presents a summary of the process drivers of changes in floods. The relative importance of individual drivers depends on the local situation and on the boundary conditions. These are discussed below, grouped into drivers related to the atmosphere, catchments, and the river system.

**Table 1:** Examples of Potential Drivers of Change in Flood Regimes and Associated Variables.^2^ (Reprinted with permission from Ref 20. Copyright 2012). Published by Copernicus Publications on behalf of the European Geosciences Union.

Compartment	Processes	Variables	Drivers of Change
Atmosphere	Atmospheric forcing of catchment water fluxes	Temperature, total precipitation, precipitation intensity/duration, snow cover and snowmelt, short‐ and long‐wave radiation climate variables	Natural climate variability at different time scales, anthropogenic climate change
Catchments	Runoff generation and concentration	Infiltration capacity, runoff coefficient, water storage capacity, evapotranspiration	Urbanization, transport infrastructure, deforestation, ditch construction, wildfires, agricultural management practices, drainage of wetlands and agricultural areas, construction of flood retention basins
Rivers	Flood wave propagation, superposition of flood waves	River morphology, conveyance, roughness, water level, runoff, floodplain storage, river channel vegetation	In‐stream channel engineering, reduction in river length, construction of dikes, groynes and weirs, operation of hydropower plants and reservoirs

### Atmosphere

The most important driver for changes in the river floods are changes in precipitation. Precipitation itself is generated by different mechanisms. Broadly speaking, large‐scale (synoptic) precipitation is related to the regional pressure distribution and the influx of atmospheric humidity. For example, winter floods in Western Europe are often due to Atmospheric Rivers, narrow ribbons along which large quantities of humidity are transported across the Atlantic from the subtropics to the mid‐latitudes.^21^ Changes in the frequency and characteristics of the global atmospheric circulation will lead to changes in the precipitation rates.^22^ Changes of these processes have been analyzed comprehensively by numerous research groups around the world, including the Intergovernmental Panel on Climate Change.^23^ Merz et al. provided a review of the state of the art.^24^ However, small‐scale (convective) precipitation tends to produce higher rainfall intensities and is related to the stability of the atmosphere (instability occurs when the lower air masses warm up due to ground heat by radiation, so their density is decreased which makes that air move up, cool down, condensate, and precipitate water). If the air is warmer, it can hold more water. The water holding capacity of the atmosphere increases by around 7% per degree of temperature increase (a relationship named after Clausius–Clapeyron). In a warmer climate, one would therefore expect higher precipitation rates. In practice, there are a number of complex processes affecting both large‐scale and small‐scale precipitation, and changes are difficult to observe. Changes in large‐scale (synoptic) precipitation are relevant in large river basins with catchment areas of hundreds to hundred thousands of square kilometres. Changes in small‐scale, convective precipitation are relevant in small catchments with areas on the order of hundred square kilometres or less.

**Figure 6:**
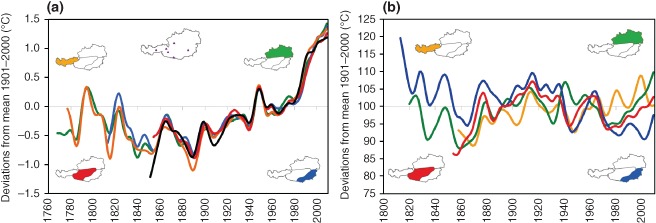
Smoothed annual series of observed (a) air temperature and (b) precipitation for subregions of Austria based on the HISTALP data set. Colors relate to different regions within Austria; black colors in (a) to a few mountain stations (Reprinted with permission from Ref 25. Copyright 2011). Publisher is Springer.

An example of the changes in observed regional precipitation is shown in Figure 6(b) for four regions in Austria. The figure suggests that, in the South of Austria, precipitation has decreased in the past 200 years (blue line). In the North of Austria, the pattern is more complex and there is a clear increase in the last two decades. Such an increase may translate in increased flood magnitudes. For comparison, Figure 6(a) shows observed air temperatures indicating clear increases in the 20th century.

**Figure 7:**
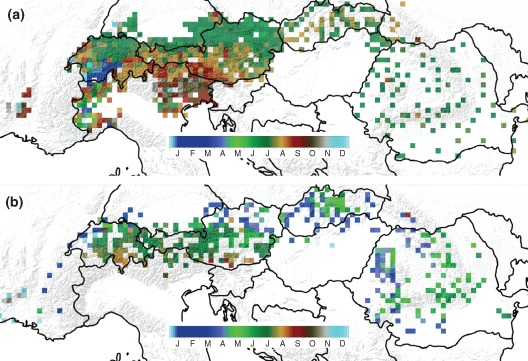
Precipitation and flood seasonality across the Alpine–Carpathian range. (a) Mean date within the year the maximum daily precipitation occurred. (b) Mean date within the year the maximum flood runoff occurred. Observed data from 1961 to 2000 (Reprinted with permission from Ref 27. Copyright 2010). Publisher is Elsevier.

Increases in air temperature are associated with changes in the energy balance of the land surface. As the air temperature increases, there is usually more energy available for melting snow, which may increase snow‐melt induced floods. It is therefore important to understand the role of snowmelt in river floods relative to the role of rainfall. There are a number of studies that have classified floods into rainfed floods, snow‐melt floods and rain‐on‐snow floods based on a detailed analysis of the processes involved.^26^ A simpler measure of the relevance of process drivers is the date of the year the floods have occurred (termed seasonality). An example for the case of the Alpine–Carpathian range is given in Figure 7. The top panel (Figure 7(a)) shows the mean date within the year the maximum daily precipitation occurred. North of the Alps, the maximum precipitation tends to occur in early summer (green color in the figure) while south of the Alps it occurs in late summer (brown color in the figure), i.e., precipitation is mainly in summer. The seasonality of the floods (Figure 7(b)) is more complex. There are summer floods in the main Alps (green and brown colors) but winter floods north of the Alps and East of the Carpathians (blue color). These differences are due to winter snowmelt and due to the role of soil moisture in producing flood runoff. Any changes in these controls will also lead to changes in the flood magnitudes.^2^

### Catchments

**Figure 8:**
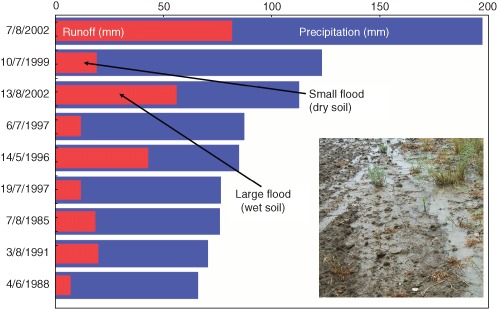
Event precipitation and runoff depths for the largest events on record in the Kamp catchment at Zwettl, Austria (Reprinted with permission from Ref 31. Copyright 2007). Published by Copernicus Publications on behalf of the European Geosciences Union.

Soil moisture is indeed a very important factor in producing flood runoff.^28^, ^29^ Soil moisture determines the amount of precipitation that cannot infiltrate and therefore runs off from the land surface and contributes to flooding. The percentage that runs off during a storm typically is between 10 and 60%^30^ and will thus be decisive for the actual magnitude of a flood. Even for the same catchment, the runoff contribution may vary vastly between events depending on the soil moisture at the beginning of that event as illustrated in Figure 8. In this particular catchment, the precipitation of the July 10, 1999 event was 120 mm (equivalent to 120 L/m^2^) out of which 20 mm ran off the surface and produced a (small) flood. This means, the contribution of precipitation to that flood was only 17%. However, the precipitation of the August 13, 2002 event was 110 mm of which 60 mm (or 55%) ran off the surface and produced a much bigger flood. The high percentage was due to a previous storm on August 7, 2002 (top bar in Figure 8). It becomes clear that, while rainfall is an important factor controlling floods, the catchment soil moisture is equally important. Any changes in the catchment soil moisture will therefore also affect the flood magnitudes.

Soil moisture is controlled by evaporation. Increases in evaporation will reduce soil moisture. However, soil moisture is also controlled by the infiltration characteristics of the soil. The more permeable a soil, the less water will accumulate near the surface. Because of this, change in land use is an important driver of flood changes. Surface sealing due to urban development will reduce infiltration. Changes in land use from agricultural land to forest (afforestation) may increase the infiltration because coarse woody roots of trees tend to create preferential flow paths in the soil (so called macropores) which enhance the fast flow of water from the surface into the subsurface, thus recharging the aquifers. Afforestation will also increase evaporation and reduce soil moisture. There is therefore a complex interplay in the soil–plant–atmosphere continuum of the processes controlling flood generation.^32^

**Figure 9:**
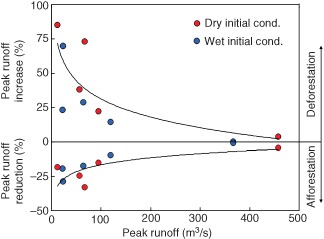
Effect of afforestation and deforestation on changes in the flood peak runoff for the 622 km^2^ Kamp catchment at Zwettl, Austria, based on rainfall‐runoff simulations. The baseline is 47% forest cover, afforestation increases it to 86%, and deforestation decreases it to 0% (remaining area is pasture and cropland). Events are stratified by the soil moisture at the beginning of the event as wet and dry (Reprinted with permission from Ref 34. Copyright 2012). Published by Copernicus Publications on behalf of the European Geosciences Union.

The role of these processes on flood generation can be assessed by paired catchment studies^33^ in which the runoff from two neighboring catchments is monitored. After a period of time, the land use in one of the catchments is changed (e.g., the forest is cut) and the resulting change in the runoff response is observed. An alternative are modeling studies that simulate the effect of land use on the flood response. A typical example of such a study is shown in Figure 9. Each point in the figure relates to one flood event. For each event, two simulations were performed, one with the real land use, and one with changed land use (either less or more forest cover). The differences in the flood peak runoff between these two simulations are plotted as percentages. The figure illustrates that afforestation tends to reduce flood peaks (which is due to increased infiltration, storage, and evaporation) while deforestation tends to increase the flood peaks. The effect is somewhat larger if the soils are dry at the beginning of the event. A scientific debate on the exact magnitudes of the effects of afforestation and deforestation on floods in different hydrological settings is ongoing.^35^, ^36^

**Figure 10:**
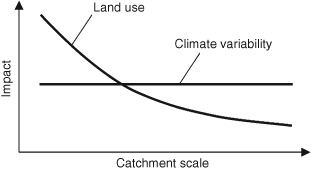
Hypothesised impact of land‐use and climate variability on flood magnitudes as a function of scale (Reprinted with permission from Ref 38. Copyright 2007). Published by John Wiley and Sons.

Similar simulation studies have been performed for large river basins.^37^ As land‐use change is usually a local phenomenon due to the rather limited spatial extent of land‐use changes, the impact of any disturbance will decrease with catchment size. In contrast, climate impacts may occur at larger scales so they will likely be relevant at both small and large scales. This scale effect is depicted schematically in Figure 10. At the level of entire river basins such as the Rhine, land‐use change effects are hardly discernable while at small scales this may well be the case.^2^ An example is urban floods at the very small scale that may be enhanced by urban development due to the reduction in the infiltration capacity of the soils.^39^

## RIVERS

As the flood waves propagate through the river system, they will be influenced by the characteristics of the channel and the flood plain. River training usually consists of straightening of the channel (to increase the flood conveyance of the channels) and construction of levees at the banks (to protect the flood plain from floods that do not exceed the crest of the levees). The straightening of the channels tends to increase the speed at which flood waves move through the system. Levees also tend to increase the wave speed as the flood waters cannot spread out onto the flood plain, so the water levels between the levees will be higher than for a case without levees. Higher water levels in turn translate into faster velocities. For example, the speed of the flood waves of the Upper Danube has increased by about 30% in the past 120 years as a result of levee construction.^40^

**Figure 11:**
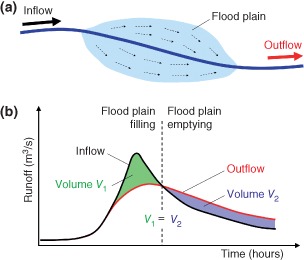
Schematic of flood plain effects on the flood hydrograph: (a) map view of a river reach with water moving from the main channel (dark blue line) out onto the flood plain and moving back to the main channel, and (b) inflow and outflow hydrograph of the reach. Flood plain retention reduces the flood peak. The magnitude of the reduction is mainly controlled by the retention volume.

Construction of river levees will also reduce the storage of flood water on the flood plain. Without levees, runoff exceeding the bank full capacity of the channel will cause the flood waters to inundate the flood plain. The volume of water in the flood plain is abstracted from the flow in the main channel which reduces the runoff. The reduction in runoff is particularly relevant if it occurs around the time of the peak runoff, as it will then reduce the peak. The processes of flood plain storage are schematically illustrated in Figure 11. The important point is that, because of mass balance, the total volume of water removed from the main channel during the rising limb of the flood is equal to that added to the main channel during the recession and that volume is equal to the storage volume in the flood plain.

Building levees will reduce this storage effect. However, removing levees or moving them further away from the river (as is done in the ‘Room for the river concept’ 41) will increase the storage effect and reduce the flood peaks. The same effect can be obtained by building polders, which is a dedicated piece of land that is allowed to flood when needed. The magnitude of the effect can be estimated by hydrodynamic models,^42^ but simple calculations can also give useful orders of magnitude. Consider, as an example, a typical flood at the Upper Danube where the flood peak runoff is around 10,000 m^3^/s (maximum of the hydrograph in Figure 11(b)). The required retention volume is the green area in the schematic in Figure 11(b). Assuming it can be approximated by a triangle of duration 2 days, and accounting for the units, a reduction by 20% of the flood peak by the construction of polders (or by removing levees) requires a total retention volume of V = 10,000 × 0.2 × 2 × 0.5 × 24 × 3600 = 1.73 × 10^8^ m^3^. If the polders are inundated by 1 m, the required area is 173 km^2^. This suggests that a lot of land area is needed for reducing flood peaks at medium sized and large rivers.

## WILL FLOODS CHANGE IN THE FUTURE?

Whether floods will occur more frequently or with bigger magnitudes in the future at a location of interest will depend on changes in the processes in the three compartments—atmosphere, catchments, and rivers. Such predictions (or sometimes called projections) are usually performed by scenario analyses where the processes driving floods are simulated by mathematical modeling. Two cases are compared:

- simulations for the current situation (without a change)
- simulations for a possible future situation (with a change)

The differences in these two simulations then give the magnitudes of the effects on the floods of changes in the drivers.

Assessing the effect of changes in the atmospheric processes starts from assumptions about the future socioeconomic conditions, say in the 21st century. From these, future greenhouse gas and aerosol emissions are estimated that are used to drive global climate models (GCMs) to estimate future greenhouse gas concentrations in the atmosphere and other climate parameters. More recently, the alternative approach of ‘Shared Socio‐Economic Pathways’ 43 has been developed where the assumptions start directly at the stage of future greenhouse gas concentrations in the atmosphere. The results of the GCM simulations are then downscaled by regional climate models, which are then used as inputs to hydrological models that simulate the flood frequencies and magnitudes.^44^

**Figure 12:**
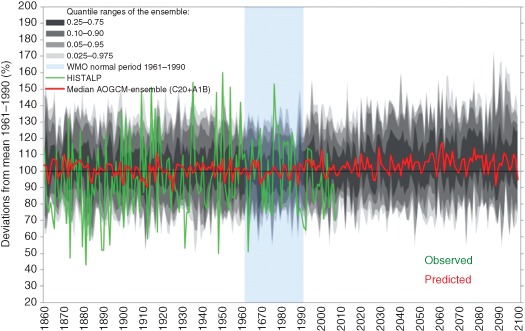
Change in the winter precipitation (December to February) from 1860 to 2100 relative to 1961–1990 in the greater Alpine area. Grey shading and red (median): 15 global ocean–atmosphere models for the IPCC SRES A1B scenario. Green: Histalp observations (Reprinted with permission from Ref 25. Copyright 2011). Published by Springer.

Figure 12 presents an example of outputs from GCMs. Shown are the differences of simulated winter precipitation in the greater Alpine area in the period 1860–2100 relative to 1961–1990. The results from fifteen GCMs have been analyzed statistically and the grey shading gives the frequency of these results. The red line shows the median of all simulations. In these simulations, natural drivers (volcano eruptions and fluctuations in solar radiation) and anthropogenic drivers (emissions) where used for the period 1860–2000 and anthropogenic drivers for 2001–2100. For comparison, the green line shows observational data of winter precipitation in the region. The between‐year variability of the simulations is much smaller than that of the observations as the former do not intend to replicate individual years but decadal variability. The figure also illustrates the uncertainties in the decadal variations of the simulations relative to the observations.

Hall et al.^2^ review the literature on future changes in floods due to climate change for the case of Europe. Some studies suggest that mean precipitation and wet‐day frequency will increase in northern Europe (translating into more frequent floods), and decrease in southern Europe (translating into less frequent floods).^45^ Additionally, regional warming may lead to increases in convective precipitation in all of Europe, which may increase floods in small catchments everywhere.

**Figure 13:**
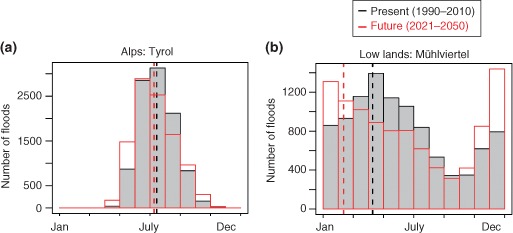
Simulated frequency of floods for two regions: (a) Tyrol and (b) Mühlviertel, Upper Austria. Current conditions (black) and scenario (red) (Reprinted with permission from Ref 48. Copyright 2011). Publisher is Springer.

Overall, predictions of changes in future extreme precipitation are less reliable than those of future seasonal (or annual precipitation), and these are less reliable than those of future air temperatures. The predictions are further complicated by scale issues.^46^ The uncertainties in the flood impact simulations are therefore usually large and, often, not fully communicated. Blöschl and Montanari therefore argue that utmost care needs to be taken in interpreting the results of such impact studies.^47^ Understanding the drivers and their changes may be more relevant than predictions of uncertain flood changes. An example of a simulation study where the focus was on the drivers is shown in Figure 13. The graphs were obtained by Monte Carlo simulations with assumptions about present and future rainfall and air temperature characteristics in two regions in Austria (Tyrol in the Alps and Mühlviertel in the low lands). In Tyrol, floods occur mainly in the summer (also see Figure 7(b), dark green colors). Under a future climate (red line in Figure 13(a)), they may occur slightly earlier in the year which is because of earlier snowmelt as a result of higher air temperatures. In the Mühlviertel region, however, floods tend to occur in spring (blue colors in Figure 7(b)) due to the wet soils in winter and spring with some snowmelt contribution. Under a future climate (red line in Figure 13(b) there is a clear shift toward more frequent winter flooding (in particular in December). This is mainly because more precipitation falls as rain (and less as snow) due to higher air temperatures. This effect is stronger than in the Alps because of the lower altitudes where snow often falls at temperatures not much below freezing.

Changes in catchment and river processes can be simulated by a similar scenario approach as that for the atmospheric processes.^49^ As, in both cases, the local situation will be the main control on any changes, such scenarios reflect the management options of flood and land management and are difficult to generalise.

The scenario approach is based on the assumption that the future system will operate similar to the past, with the exception to the one quantity that is changed (e.g., changed climate, land use, or levees).^50^ However, Sivapalan et al. argued that this may not be a very realistic assumption if one predicts decades or centuries into the future.^51^ Any changes in the hydrological system will also affect the socioeconomic system and vice versa. They coined the term sociohydrology as the science that considers humans as an integral part of the entire system. The idea is to go beyond the quasi‐stationarity of the scenario approach (as assumed in Figure 13, e.g.) and focus on feedbacks of the long‐term dynamics. The system components (society, infrastructure, and catchments) may coevolve over long time periods because they are connected.

**Figure 14:**
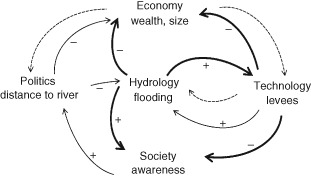
Loop diagram showing how hydrological, economical, political, technological, and social processes are all interlinked and gradually (continuous thin arrows) coevolve, while being abruptly (continuous thick arrows) altered by the sudden occurrence of flooding events. Dashed arrows indicate more indirect control mechanisms (Reprinted with permission from Ref 18. Copyright 2013). Published by Copernicus Publications on behalf of the European Geosciences Union.

To illustrate the role of feedbacks for river floods, Di Baldassarre et al. and Viglione et al. proposed a simple (stylised) model that represents the interplay of the main processes associated with floods for a hypothetical city located at a river.^18^, ^52^ Figure 14 shows the loop diagram of the model. Each arrow represents a connection between the components. The components are the economy (in terms of wealth of the city), the technology (in terms of level of flood protection), the hydrology (in terms of flood magnitudes and damage), politics (in terms of urban planning), and society (in terms of risk awareness). In the model, each component is represented by a dynamic differential equation. The equations are nonlinear and coupled. The model does not represent one particular city. Instead, it can be used to explore the general feedbacks of such a system.

**Figure 15:**
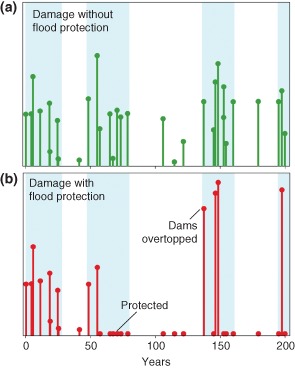
Two scenarios of flood damage for a hypothetical city: (a) flood management options involve the choice of building close or far away of flood prone river but no levees and (b) flood management options also involve the construction of levees. Light blue areas indicate flood‐rich periods, white areas flood‐poor periods. Results from the sociohydrology model of Refs 18 and 52.

An example of the simulation result of the model is shown in Figure 15. In the simulations, time series of 200 years of floods (similar to those in Figure 2) were assumed. The coupled model simulates the evolution of the city over these two centuries including whether citizens decide to build close to the river (which has economic advantages) or far away from the river (which tends to avoid flood damage), and they may decide to build levees to protect them from flooding (but these may be overtopped). In Figure 15, there are two scenarios. At the top (Figure 15(a)), the flood management options involve the choice of building close or far away of flood prone river but no levees. At the bottom (Figure 15(b)), the flood management options also involve the construction of levees. Without flood protection, each flood results in a commensurate flood damage. During the flood‐rich periods, there is of course more flood damage than in the flood‐poor periods. For the case where flood protection (i.e., levees) is gradually constructed in the model (mainly around year 50 in bottom scenario), the damage is significantly reduced because of the protection (as compared with the scenario at the top). However, during the ensuing flood poor period (years 80–130) (when the floods are smaller than the protection level and therefore do not cause any damage) citizens tend to forget about the flood risk and settle close to the levees. Once the flood‐rich period sets in, the damage is larger than for the case without flood protection. While these are hypothetical scenarios, they do underline that feedbacks between the components of the sociohydrological system may be very important. Of course, even without human interventions, hydrological processes feedback with underlying natural causes in the atmosphere, the landscape, and the river system over long time scales, thus influencing river floods, e.g., as conceptualised in Perdigão and Blöschl.^29^

Flood management, ideally should account for all these complexities using local information in a river basin context. Many countries have recently passed legislation such as the EU Flood directive^53^ that explicitly requires the establishment of flood risk management plans. The overall idea is that the flood risk problem is dealt with in a comprehensive way; hence the term integrated flood risk management (IFRM) is currently used. The term ‘integrated’ refers to the integration between sectors (such as water management, transport, regional planning, and tourism) and between upstream and downstream reaches in a river basin.

Risk is usually conceptualised to consist of two components: hazard and vulnerability. The hazard relates to the characteristics of the flood while the vulnerability relates to the characteristics of the people, the property or the environment that are at risk. Risk management may either reduce the hazard (e.g., by building polders), or the vulnerability (e.g., by building further away from the river), or both. In fact, IFRM may involve a wide range of measures, including structural measures such as levees for flood protection, polders of flood mitigation, and nonstructural measures such as land‐use zoning and insurances. IFRM may also increase the preparedness by establishing emergency plans, training flood management staff, awareness building of the general public, and flood warnings. Once a flood has occurred, the response may involve evacuations and the provision of food and shelter. Longer‐term responses may include cleanup and rebuilding of structures.^54^

There are two fundamental approaches to risk management. The first is termed the ‘predict‐then‐act’ method where future flood risk is estimated which is then used as the basis for choosing among alternative flood management options.^55^ The future risk is estimated by combining the future hazard (e.g., estimated by downscaling GCM simulations to drive hydrological models) with the future vulnerability (e.g., estimated by assessing the potential damage). The second, alternative, approach is the vulnerability approach. It starts at the local scale of individuals, households, and communities, and explores the factors and conditions that enable successful coping with flood risk.^56^, ^57^ This approach also works for situations where the individual factors contributing to the flood risk are not know in detail or cannot be anticipated. For example, Blöschl et al. report on a dam failure that resulted from the inability of the dam managers to open the flood gates as they had blocked them previously because of concerns about sabotage.^58^ This is an example of a situation that can be hardly anticipated by engineers planning a dam.

**Figure 16:**
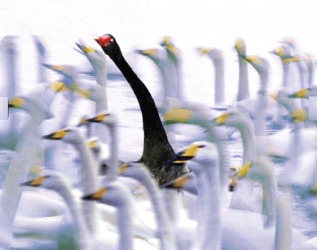
A black swan among numerous white swans is unexpected but may be the important one. There is a potential for Black Swan events in hydrology that are unexpected but have high impact from a societal point of view. Available at: http://www.lonelyplanet.com/japan/hokkaido/images/black‐swan‐among‐white‐swans‐hokkaido$24256‐1.

These types of unexpected or surprising events may be more widespread than what one usually thinks. Taleb^59^ termed such unexpected, large impact events ‘Black Swan events’, based on the anecdote that, before the discovery of Australia, all swans were considered to be white because of the lack of black swan sightings in the western world (Figure 16). The 2001 terrorist attack on the World Trade Centre is an example of a Black Swan event. For such events, prior risk calculations will be grossly in error. Taleb^59^ also noted that the worst disasters in history have been the unexpected ones because of the inability to brace against them. The vulnerability approach is suitable for dealing with Black Swan events as there is no need to specify the causes of the flood risk. An example of implementing such a strategy has been reported by Wardekker et al.^60^ who explored imaginable surprises (termed ‘wildcards’) when proposing a flood risk management strategy for the city of Rotterdam. Blöschl et al.^58^ argue that a combination of ‘predict‐then‐act’ and the vulnerability approaches will often be in order. The latter gains in importance for very large potential societal consequences of floods at the location of interest.

## CONCLUSIONS

This article has explored whether floods have changed in the past. In Europe, the magnitude and frequency of floods have indeed changed in a complex manner with flood‐rich and flood‐poor periods alternating. In some parts of Europe, the present may be a flood‐rich period. While increasing river flood runoff is reality rather than fiction at some locations, depending on their influencing factors, in many regions the socioeconomic conditions have changed even more, resulting in increases in flood damage.

This article has also examined the driving processes of change. Changes in the frequency and characteristics of the global atmospheric circulation will lead to changes in regional precipitation, and warming may lead to higher convective precipitation. These changes are very significantly modulated by soil moisture and snowmelt processes. Land‐use change effects (such as deforestation and urbanisation) on floods may be important for small catchments, but at the large river basin scale, these effects are usually very small. River training and the construction of polders may also affect the flood magnitudes. The main parameter controlling the flood peak is the flood retention volume available on the flood plain.

Future changes in the frequency and magnitudes of floods at a particular location will depend on changes in the three compartments—atmosphere, catchments, and rivers. Scenario methods are used to explore such changes. Future air temperatures can be anticipated with less uncertainty than future extreme precipitation. Because of this, flood change predictions associated with snowmelt are more reliable than those associated with heavy precipitation. When lead times of decades or centuries are of interest, it is important to account for the long term feedbacks between the hydrological system and the societal system by coupling fully the system components as is done in sociohydrology. For high‐risk situations, unexpected extreme events (so called Black Swan events) are important. To account for such events, the vulnerability‐based approach to IFRM should be combined with the traditional ‘predict‐then‐act’ method.

## References

[1] Viglione A , Rogger M . Flood processes and hazards In: Paron P, Di Baldassarre G, Shroder JF Jr, eds. Hydro‐Meteorological Hazards, Risks, and Disasters. Amsterdam: Elsevier; 2015.

[2] Hall J , Arheimer B , Borga M , Brázdil R , Claps P , Kiss A , Kjeldsen TR , Kriaučiūnienė J , Kundzewicz ZW , Lang M , et al. Understanding flood regime changes in Europe: a state of the art assessment. Hydrol Earth Syst Sci 2014, 18:2735–2772. doi:10.5194/hess-18-2735-2014.

[3] Blöschl G , Nester T , Komma J , Parajka J , Perdigão RAP . Das Juni‐Hochwasser 2013—Analyse und Konsequenzen für das Hochwasserrisikomanagement (The June 2013 flood—analysis and implications for flood risk management). Z Österreich Ingen‐&‐Architekten‐Ver 2013, 158:141–152.

[4] Kundzewicz ZW, ed. Changes in Flood Risk in Europe. IAHS Special Publ. No. 10 Wallingford: IAHS Press; 2012, 516 + xvi pp.

[5] Brázdil R , Kundzewicz ZW , Benito G . Historical hydrology for studying flood risk in Europe. Hydrol Sci J 2006, 51:739–764.

[6] Brázdil R , Kundzewicz ZW , Benito G , Demarée G , Macdonald N , Roald LA . Historical floods in Europe in the Past Millennium In: Kundzewicz ZW, ed. Changes in Flood Risk in Europe. Wallingford: IAHS Press; 2012, 121–166.

[7] Kjeldsen TR , Macdonald N , Lang M , Mediero L , Albuquerque T , Bogdanowicz E , Brázdil R , Castellarin A , David V , Fleig A , et al. Documentary evidence of past floods in Europe and their utility in flood frequency estimation. J Hydrol 2014, 517:963–973.

[8] Baker VR , Webb RH , House PK . The scientific and societal value of paleoflood hydrology In: House PK, Webb RH, Baker VR, Levish DR, eds. Ancient Floods, Modern Hazards: Principles and Applications of Paleoflood Hydrology, vol. 5 Washington, DC: American Geophysical Union, Water Science and Application Series; 2002, 127–146.

[9] Kiss A , Laszlovszky J . 14th–16th‐century Danube floods and long‐term waterlevel changes in archaeological and sedimentary evidence in the western and central Carpathian Basin: An overview with documentary comparison. J Environ Geogr 2013, 6:1–11.

[10] Schmocker‐Fackel P , Naef F . Changes in flood frequencies in Switzerland since 1500. Hydrol Earth Syst Sci 2010, 14:1581–1594.

[11] Hurst HE . Long term storage capacity of reservoirs. Trans Am Soc Civil Eng 1951, 116:770–779.

[12] Montanari A , Taqqu MS , Teverovsky V . Estimating long‐range dependence in the presence of periodicity: an empirical study. Math Comput Model 1999, 29:217–228.

[13] Szolgayová E , Laaha G , Blöschl G , Bucher C . Factors influencing long range dependence in streamflow of European rivers. Hydrol Process 2013, 28:1573–1586. doi:10.1002/hyp.9694.

[14] Pires CAL , Perdigão RAP . Non‐Gaussian interaction information: estimation, optimization and diagnostic application of triadic wave resonance. Nonlin Processes Geophys Discuss 2014, 1:1539–1602. doi:10.5194/npgd-1-1539-2014.

[15] Huntingford C , Marsh T , Scaife AA , Kendon EJ , Hannaford J , Kay AL , Lockwood M , Prudhomme C , Reynard NS , Parry S , et al. Potential influences on the United Kingdom's floods of winter 2013/14. Nat Clim Change 2014, 4:769–777. doi:10.1038/nclimate2314.

[16] Merz B , Kreibich H , Schwarze R , Thieken A . Review article: assessment of economic flood damage. Nat Hazards Earth Syst Sci 2010, 10:1697–1724.

[17] Barredo JI . Normalised flood losses in Europe: 1970–2006. Nat Hazards Earth Syst Sci 2009, 9:97–104.

[18] Di Baldassarre G , Viglione A , Carr G , Kuil L , Salinas JL , Blöschl G . Socio‐hydrology: conceptualising human‐flood interactions. Hydrol Earth Syst Sci 2013, 17:3295–3303. doi:10.5194/hess-17-3295-2013.

[19] Montanari A , Young G , Savenije HHG , Hughes D , Wagener T , Ren LL , Koutsoyiannis D , Cudennec C , Toth E , Grimaldi S , et al. “Panta Rhei—everything flows”: change in hydrology and society—The IAHS scientific decade 2013–2022. Hydrol Sci J 2013, 58:1256–1275.

[20] Merz B , Vorogushyn S , Uhlemann S , Delgado J , Hundecha Y . HESS opinions—more efforts and scientific rigour are needed to attribute trends in flood time series. Hydrol Earth Syst Sci 2012, 16:1379–1387.

[21] Lu M , Lall U , Schwartz A , Kwon H . Precipitation predictability associated with tropical moisture exports and circulation patterns for a major flood in France in 1995. Water Resour Res 2013, 49:6381–6392.

[22] Pires CAL , Perdigão RAP . Non‐Gaussianity and asymmetry of the winter monthly precipitation estimation from the NAO. Mon Weather Rev 2007, 135:430–448. doi:10.1175/MWR3407.10.

[23] IPCC . Summary for policymakers In: Stocker TF, Qin D, Plattner G‐K, Tignor M, Allen SK, Boschung J, Nauels A, Xia Y, Bex V, Midgley PM, eds. Climate Change 2013: The Physical Science Basis. Contribution of Working Group I to the Fifth Assessment Report of the Intergovernmental Panel on Climate Change. Cambridge, UK: Cambridge University Press; 2013.

[24] Merz B , Aerts J , Arnbjerg‐Nielsen K , Baldi M , Becker A , Bichet A , Blöschl G , Bouwer LM , Brauer A , Cioffi F , et al. Floods and climate: emerging perspectives for flood risk assessment and management. Nat Hazards Earth Syst Sci 2014, 14:1921–1942. doi:10.5194/nhess-14-1921-2014.

[25] Schöner W , Böhm R , Haslinger K . Climate change in Austria—climate variables of hydrological relevance (Klimaänderung in Österreich—hydrologisch relevante Klimaelemente (in German)). Österr Wasser‐Abfallwirtsch 2011, 63:11–20.

[26] Merz R , Blöschl G . A process typology of regional floods. Water Resour Res 2003, 39:1340–1359.

[27] Parajka J , Kohnová S , Bálint G , Barbuc M , Borga M , Claps P , Cheval S , Dumitrescu A , Gaume E , Hlavová K , et al. Seasonal characteristics of flood regimes across the Alpine–Carpathian range. J Hydrol 2010, 394:78–89. doi:10.1016/j.jhydrol.2010.05.015. 10.1016/j.jhydrol.2010.05.015 PMC4106690 25067854

[28] Rogger M , Viglione A , Derx J , Blöschl G . Quantifying effects of catchments storage thresholds on step changes in the flood frequency curve. Water Resour Res 2013, 49:6946–6958. doi:10.1002/wrcr.20553.

[29] Perdigão RAP , Blöschl G . Spatiotemporal flood sensitivity to annual precipitation: evidence for landscape‐climate coevolution. Water Resour Res 2014, 50:5492–5509. doi:10.1002/2014WR015365.

[30] Merz R , Blöschl G . A regional analysis of event runoff coefficients with respect to climate and catchment characteristics in Austria. Water Resour Res 2009, 45:W01405. doi:10.1029/2008WR007163.

[31] Komma J , Reszler C , Blöschl G , Haiden T . Ensemble prediction of floods—catchment non‐linearity and forecast probabilities. Nat Hazards Earth Syst Sci 2007, 7:431–444.

[32] Gaál L , Szolgay J , Kohnová S , Parajka J , Merz R , Viglione A , Blöschl G . Flood timescales: understanding the interplay of climate and catchment processes through comparative hydrology. Water Resour Res 2012, 48:W04511.

[33] Brown AE , Zhang L , McMahon TA , Western AW , Vertessy RA . A review of paired catchment studies for determining changes in water yield resulting from alterations in vegetation. J Hydrol 2005, 310:28–61.

[34] Salazar S , Francés F , Komma J , Blume T , Francke T , Bronstert A , Blöschl G . A comparative analysis of the effectiveness of flood management measures based on the concept of "retaining water in the landscape" in different European hydro‐climatic regions. Nat Hazards Earth Syst Sci 2012, 12:3287–3306. doi:10.5194/nhess-12-3287-2012.

[35] Jones JA , Grant GE . Peak flow responses to clear‐cutting and roads in small and large basins, western Cascades, Oregon. Water Resour Res 1996, 32:959–974.

[36] Beschta RL , Pyles MR , Skaugset AE , Surfleet CG . Peakflow responses to forest practices in the western cascades of Oregon, USA. J Hydrol 2000, 233:102–120.

[37] Bronstert A , Niehoff D , Bürger G . Effects of climate and land‐use change on storm runoff generation: present knowledge and modelling capabilities. Hydrol Process 2002, 16:509–529.

[38] Blöschl G , Ardoin‐Bardin S , Bonell M , Dorninger M , Goodrich D , Gutknecht D , Matamoros D , Merz B , Shand P , Szolgay J . At what scales do climate variability and land cover change impact on flooding and low flows? Hydrol Process 2007, 21:1241–1247.

[39] Kennedy JR , Goodrich DC , Unkrich CL . Chapter 11.16: model enhancements for urban runoff predictions in the south‐west USA In: Blöschl G, Sivapalan M, Wagener T, Viglione A, Savenije HHG, eds. Runoff Prediction in Ungauged Basins: Synthesis across Processes, Places and Scales. Cambridge: Cambridge University Press; 2013, 332–337.

[40] Blöschl G , Nester T , Komma J , Parajka J , Perdigão RAP . The June 2013 flood in the Upper Danube basin, and comparisons with the 2002, 1954 and 1899 floods. Hydrol Earth Syst Sci 2013, 17:5197–5212.

[41] Rijke J , van Herk S , Zevenbergen C , Ashley R . Room for the river: delivering integrated river basin management in the Netherlands. Int J River Basin Manage 2012, 10:369–382.

[42] Skublics D , Rutschmann P . Progress in natural flood retention at the Bavarian Danube. Nat Hazards 2013, 75:1–17. doi:10.1007/s11069-014-1148-x.

[43] van Vuuren DP , Edmonds J , Kainuma M , Riahi K , Thomson A , Hibbard K , Hurtt GC , Kram T , Krey V , Lamarque J‐F , et al. The representative concentration pathways: an overview. Clim Change 2011, 109:5–31.

[44] Raff D , Pruitt T , Brekke L . A framework for assessing flood frequency based on climate projection information. Hydrol Earth Syst Sci 2009, 13:2119–2136.

[45] Rajczak J , Pall P , Schär C . Projections of extreme precipitation events in regional climate simulations for Europe and the Alpine region. J Geophys Res Atmos 2013, 118:3610–3626. doi:10.1002/jgrd.50297.

[46] Blöschl G , Sivapalan M . Scale issues in hydrological modelling: a review. Hydrol Process 1995, 9:251–290.

[47] Blöschl G , Montanari A . Climate change impacts ‐ throwing the dice? Hydrol Process 2010, 24:374–381. doi:10.1002/hyp.7574.

[48] Blöschl G , Viglione A , Merz R , Parajka J , Salinas JL , Schöner W . Climate impacts on floods and low flows (Auswirkungen des Klimawandels auf Hochwasser und Niederwasser (in German)). Österr Wasser‐Abfallwirtsch 2011, 63:21–30.

[49] Apel H , Thieken AH , Merz B , Blöschl G . A probabilistic modelling system for assessing flood risks. Nat Hazards 2006, 38:79–100.

[50] Peel MC , Blöschl G . Hydrologic modelling in a changing world. Prog Phys Geogr 2011, 35:249–261.

[51] Sivapalan M , Savenije HHG , Blöschl G . Socio‐hydrology: a new science of people and water. Hydrol Process 2012, 26:1270–1276. doi:10.1002/hyp.8426.

[52] Viglione A , Di Baldassarre G , Brandimarte L , Kuil L , Carr G , Salinas JL , Scolobig A , Blöschl G . Insights from socio‐hydrology modelling on dealing with flood risk—roles of collective memory, risk‐taking attitude and trust. J Hydrol 2014, 518:71–82.

[53] European Union . Directive 2007/60/EC of the European Parliament and of the Council of 23 October 2007 on the assessment and management of flood risks. Official Journal of the European Union, L 288/27‐34; 2007.

[54] Thieken AH , Kreibich H , Müller M , Merz B . Coping with floods: preparedness, response and recovery of flood‐affected residents in Germany in 2002. Hydrol Sci J 2007, 52:1016–1037.

[55] Dessai S , Hulme M . Does climate adaptation policy need probabilities? Clim Policy 2004, 4:107–128.

[56] Wilby RL , Dessai S . Robust adaptation to climate change. Weather 2010, 65:180–185.

[57] Wilby RL , Keenan R . Adapting to flood risk under climate change. Prog Phys Geogr 2012, 36:349–379.

[58] Blöschl G , Viglione A , Montanari A . Emerging approaches to hydrological risk management in a changing world In: Pielke R, ed. Climate Vulnerability: Understanding and Addressing Threats to Essential Resources. Amsterdam: Academic Press, Elsevier Inc.; 2013, 3–10.

[59] Taleb NN . The Black Swan: The Impact of the Highly Improbable. New York City: Random House; 2007.

[60] Wardekker JA , de Jong A , Knoop JM , van der Sluijs JP . Operationalising a resilience approach to adapting an urban delta to uncertain climate changes. Technol Forecast Soc Change 2010, 77:987–998. doi:10.1016/j.techfore.2009.11.005.

